# Angiogenesis and Progression in Human Melanoma

**DOI:** 10.1155/2010/185687

**Published:** 2010-06-06

**Authors:** R. Ria, A. Reale, A. Castrovilli, G. Mangialardi, F. Dammacco, D. Ribatti, A. Vacca

**Affiliations:** ^1^Department of Biomedical Sciences and Human Oncology, Section of Internal Medicine and Clinical Oncology, University of Bari Medical School, Bari I-70124, Italy; ^2^Department of Human Anatomy and Histology, University of Bari Medical School, Bari I-70124, Italy

## Abstract

In tumor growth, angiogenesis, the process of new-formation of blood vessels from pre-existing ones, is uncontrolled and unlimited in time. The vascular phase is characterized by the new-formation of vascular channels that enhances tumor cell proliferation, local invasion and hematogenous metastasis. Human malignant melanoma is a highly metastatic tumor with poor prognosis, and high resistance to treatment. Parallel with progression, melanoma acquires a rich vascular network, whereas an increasing number of tumor cells express the laminin receptor, which enables their adhesion to the vascular wall, favouring tumor cell extravasation and metastases. Melanoma neovascularization has been correlated with poor prognosis, overall survival, ulceration and increased rate of relapse. Secretion of various angiogenic cytokines, i.e. VEGF-A, FGF-2, PGF-1 and -2, IL-8, and TGF-1 by melanoma cells promote the angiogenic switch and has been correlated to transition from the radial to the vertical growth phase, and to the metastatic phase. Moreover, melanoma cells overexpress αvβ3, αvβ5, α2β1 and α5β1 integrins and release, together with stromal cells, higher amount of metalloproteases that increasing their invasive potential and angiogenesis. Basing on these observations, different molecular targets of antiangiogenic molecules has be recognized and various antiangiogenic agents are currently in preclinical and clinical trials for melanoma.

## 1. Introduction

Angiogenesis, the process of new formation of blood vessels from preexisting ones, takes place in both physiological and pathological conditions, such as chronic inflammation and cancer [1, 2]. In tumor growth, angiogenesis is uncontrolled and unlimited in time and it is involved in the transition from the avascular to the vascular phase [3], the so-called angiogenic switch, in which the balance between angiogenesis inducers and inhibitors leans towards the former [4]. The vascular phase is characterized by the new formation of vascular channels that enhance tumor cell proliferation, local invasion, and hematogenous metastasis. 

## 2. Angiogenesis in Human Melanoma

Human malignant melanoma is a highly metastatic tumor with poor prognosis and high resistance to treatment. It progresses through different steps: nevocellular nevi, dysplastic nevi (when these two entity can be identified as primary events in melanocytic neoplasia progression), in situ melanoma, radial growth phase melanoma (Breslow index ≤0.75 mm), vertical growth phase melanoma (index >0.75 mm), and metastatic melanoma [5]. Primary tumor grows horizontally through the epidermis; over time, a vertical growth phase component intervenes and melanoma increases its thickness and invades the dermis. Once a vertical growth phase has developed, there is a direct correlation between the tumor thickness and the number of metastases [6]. 

Parallel with progression, melanoma acquires a rich vascular network, whereas an increasing number of tumor cells express the laminin receptor, which enables their adhesion to the vascular wall, favouring tumor cell extravasation and metastases [7–9]. Melanoma neovascularization has been correlated with poor prognosis, overall survival, ulceration, and increased rate of relapse [10–12].

## 3. The Role of Angiogenic Cytokines

Secretion of vascular endothelial growth factor-A (VEGF-A) by melanoma cells has been correlated to the transition from the radial to the vertical growth phase, and to the metastatic phase [13–15]. Ribatti et al. [12] have demonstrated that increased microvascular density, strong VEGF-A tumor immunoreactivity, increased vascular diameter, and high number of vascular pillars—expression of the intussusceptive microvascular growth—are correlated to a high Breslow index (>3.6 mm). Salven et al. [15] have demonstrated that up-regulation of VEGF-A expression in metastatic melanoma is associated with an increase in the number of tumor-infiltrating inflammatory cells expressing VEGF-A. Finally, melanotransferrin, which is angiogenic in vitro and in vivo, is overexpressed in human melanoma and correlates to the tumor VEGF-A expression and progression [16].

Fibroblast growth factor-2 (FGF-2) is overexpressed in human melanoma and may be induced by an increased release by tumor cells of matrix metalloproteinases (MMPs) which, in turn, degrade extracellular matrix inducing the release of FGF-2 stored there as an inactive form. Ribatti et al. [17] have demonstrated a significant correlation between melanoma progression, percentage of FGF-2-expressing tumor cells, and the number of mast cells which, in turn, secrete other angiogenic molecules, such as VEGF-A [15].

Another important stimulator of melanoma angiogenesis is placental growth factor (PGF). PGF-1 and -2 are expressed by melanoma cells and known to bind neuropilin-1 and -2 receptors expressed on endothelial cells [18]. In addition, PGF acts through binding to VEGF receptor-1 inducing the mobilization and recruitment of VEGFR-1+ hematopoietic precursors from bone marrow and enhancing blood vessel maturation by acting on VEGFR-1-expressing smooth muscle cells/pericytes [19]. Moreover, PGF forms heterodimers with VEGF-A and enhances melanoma angiogenesis by activating VEGFR-2 on endothelial cells [19, 20]. 

Interleukin-8 (IL-8) expression was found to be very little in normal epidermis and benign melanocytic lesions. However, it is dramatically increased in a majority of cutaneous melanomas. Its serum levels in patients are significantly elevated compared to healthy individuals and correlate with advanced disease stage as well as with overall survival [21]. Melanoma-derived IL-8 is able to induce endothelial cell migration, modulate vascular permeability, and enhance actin stress fiber formation. These activities resulted in enhanced angiogenesis, rapid tumor growth, and increased metastatic potential [22, 23]. Liu et al. [24] have demonstrated that transforming growth factor-1 (TGF-1) is able to enhance expression of IL-8 in human melanoma cells and promote angiogenesis in several mouse xenograft models.

## 4. Integrin Signaling and Extracellular Matrix Enzymes

Vacca et al. [7, 9] have demonstrated that melanoma cells express the 67-kDa laminin receptor in step with the progression from the nevocellular to the dysplastic nevi, and from the primary to the metastatic tumor. This expression enables melanoma cell adhesion to the vascular wall and together with the increased vascular network favors tumor cell extravasation and metastasis. 

Overexpression of *α*v*β*3, *α*v*β*5, *α*2*β*1, and *α*5*β*1 integrins has been correlated with the transition from primary to metastatic melanoma [25]. In turn, integrins overexpression stimulates MMP-2 and MMP-7 in melanoma cells, increasing their invasive potential [26].

Melanoma and tumor stromal cells express several MMPs, including MMP-1, -2, -3, -7, -9, -14, -15, -16, as well as tissue inhibitors of MMPs such as TIMP-1, -2, and -3 [27]. MMPs overexpression has been correlated with increased microvascular density, Bcl-2 overexpression, and low survival rate. The most extensively studied MMPs in melanomas are MMP-2 and MMP-9. The expression and activation of both enzymes have been correlated to the invasive and metastatic phenotypes of the tumors [27–31] in which they are constitutively expressed and highly associated with atypia and dedifferentiation into melanocytic lesions [28]. MMP-2 expression was highly correlated with the metastatic spread and low survival rates [27]. Moreover, functional activity of MMPs is required for tumor progression. Overexpression of MT1-MMP in melanoma cells induced activation of MMP-2 which is crucial for extracellular matrix degradation. MMP-2 and MT1-MMP+ tumor cells were often restricted to the interface between the tumor invasive part and stroma [32, 33]. Expression of MMPs is not restricted to tumor cells but is also found abundantly in stromal cells indicating a major contribution of host-derived proteases to tumor progression [34]. Also MMP-1 expression is highly associated with melanoma progression [29]. MMP-9 expression in melanoma cells was found exclusively during the horizontal growth phase but not during the vertical phase. This clearly suggests that expression of MMP-9 is an early event in melanoma progression [28]. 

Several studies using either cell lines or animal models have demonstrated that the balance between MMPs and their inhibitors (TIMPs) finally determines melanoma progression [33–39]. Overexpression of TIMP-1, -2, and -3 significantly reduces melanoma tumor cell invasion, migration, growth and metastasis, and significantly reduces tumor neovascularization in the several tumor models studied [40]. 

Urokinase plasminogen activator and its receptor (uPA/uPAR) have been demonstrated to play a crucial role in several stages of melanoma progression including tumor cell migration, invasion, and metastasis. uPA secreted from melanoma cells is able to regulate endothelial cell functions including migration and the organization of endothelial cells into tube-like structures [41–43]. 

The extracellular matrix enzymes and their inhibitors play also an important role in cancer dysregulated angiogenesis [44, 45]. These enzymes are the major degrading enzymes produced by angiogenic endothelial cells for migration trough extracellular matrix during neovessel formation [46]. Moreover, MMPs and TIMPs may act as regulators of signaling pathways through the cleavage of nonmatrix substrates, including cytokines, chemokines, and growth factors. In the last fifteen years, different extracellular matrix proteins and cleavage products have been identified. These molecules possess the ability to regulate vascular development, repair and function. Therefore, possible regulatory mechanisms in vascular biology controlled by different cleavage products of basement membrane proteins (e.g., endostatin and tumstatin, endorepellin), their activation by proteases and inhibitors, such as matrix metalloproteases (MMPs), cathepsins, tissue inhibitors of MMPs and cystatin, will be reviewed [47].

## 5. Antiangiogenic Therapies

As it is shown on Table 1, different molecular targets of antiangiogenic molecules can be recognized, so various antiangiogenic agents are currently in clinical trials for melanoma.

Thalidomide has been found to have antiangiogenesis and antiinflammatory properties, and accordingly it has been used as a therapeutic agent in some malignant tumors including liver, renal cell, and breast carcinomas [48]. Thalidomide inhibits vasculogenic mimicry channel and mosaic vessels formation in melanoma through the regulation of vasculogenic factors, and it can induce necrosis of tumor cells, which may be related with the NF-kappaB signaling pathway [49, 50]. Many studies are also focused on the effects of thalidomide on advanced melanoma alone [51, 52] or in combination with Interferon alpha 2 b [53, 54], temozolomide [55, 56], and dacarbazine [57] with encouraging results. 

On the basis of preclinical findings indicating that continuous low dose (metronomic) chemotherapy is thought to inhibit tumor angiogenesis, [58] the evaluation of antiangiogenic potency of various chemotherapeutic drugs for metronomic chemotherapy, particularly taxol, is ongoing for its efficacy.

Semaxanib, a small molecule inhibitor of the VEGFR-2 tyrosine kinase, has shown encouraging results in patients with metastatic melanoma [59, 60] in whom it has also been evaluated in combination with thalidomide to assess the efficacy, tolerability, pharmacokinetic, and pharmacodynamic characteristics [61]. The results of this last study have demonstrated that the combination semaxanib-thalidomide is feasible and demonstrated antitumor activity in patients with metastatic melanoma who had failed prior therapy.

Another way to inhibit angiogenesis is the inhibition of matrix metalloproteinase (MMP) activity. In the early 1990, MMP inhibitors generated great enthusiasm among several research groups wishing to take them to clinical trials. Preclinical trials of MMP inhibitors were very promising, showing minimum side effects compared to other drugs available at the time. Several current inhibitors, which have been tested in preclinical and clinical trials, are broad category MMP inhibitors [62–64]. Pharmacological inhibitors such as prinomastat, batimastat, and its analog marimastat, which interfere with the catalytic site of the MMPs, were the first inhibitors studied in detail. Most of the inhibitors tested in clinical trials were not very promising due to the lack of positive outcomes and the appearance of substantial drug side effects, which were not observed in preclinical studies. Therefore, most of the inhibitor clinical trials were terminated following phase 3 clinical trials [63, 64]. 

Good therapeutic effects have been obtained in little studies with the combination of bevacizumab (the anti-VEGF monoclonal antibody) and chemotherapy in advanced melanoma [65–68]. Moreover, preclinical data strongly support the use of a combination of bevacizumab and erlotinib, a tyrosine kinase receptors inhibitor [67].

PI-88, a potent inhibitor of heparanase, demonstrates an overall survival and time to progression similar to standard chemotherapy [69].

Preclinical data suggest that the ectopic expression of alphaIIb beta3 in melanoma cells can be exploited as a novel target of antibody therapy [70]. 

Although most of these study have obtained encouraging results, further evaluations of therapeutic strategies that target multiple angiogenesis pathways may be warranted in patients with advanced melanoma and other malignancies.

Finally, antiangiogenesis therapy might have the unintended effect of promoting tumor metastasis by increasing vasculogenic mimicry as an alternative circulatory system [71]. When the endothelium-dependent vessels are inhibited by the effective angiogenesis inhibitors, the hypoxia of tumor cells caused by antiangiogenesis may increase vasculogenic mimicry compensatively which can replace the job of endothelium-dependent vessels to maintain the tumor blood supply and provide a convenient route of tumor metastasis. As a result, antiangiogenesis therapy might have the unintended effect of promoting tumor metastasis by increasing vasculogenic mimicry.

## 6. Concluding Remarks

**Figure 1:**
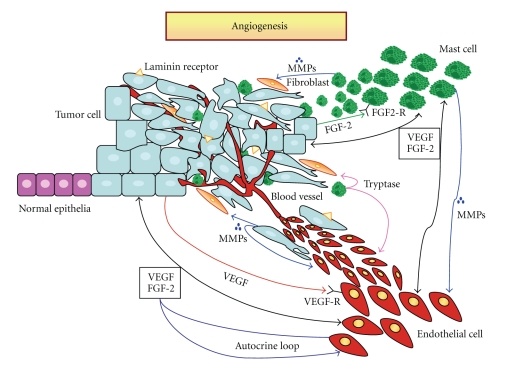


Angiogenesis in melanoma is crucial for tumor progression and metastasic escape. Since this process involves a synergistic action of several classes of angiogenic molecules and signaling pathways (Figure 1), several possibilities exist for the development of antiangiogenic therapeutic strategies. Numerous angiostatic compounds are already in clinical trials, but these approaches should be further developed.

## Figures and Tables

**Table 1:** Molecular targets of antiangiogenic drugs.

Category	Molecular Targets	Name
Angiogenic growth factors and their receptors	VEGF	Bevacizumab
	Tyrosine kinase receptors	Sorafenib
	VEGF receptors	PTK/ZK
		DC101

Receptors for extracellular matrix, integrins	Integrin *α*v*β*3	Vitaxin (MEDI-52)
	*α*v*β*3/*α*v*β*5	Cilengitide
		(EMD 121974)

Components of extracellular matrix and proteases	MMPs	Batimastat
		Marimastat
	Extracellular matrix	Endostatin

Complex mechanism of action	Angiogenesis inhibitors and immunomodulators	Thalidomide
		Lenalidomide
	Cyclooxygenase-2	Celecoxib
